# Genomic imbalances in 70 snap-frozen cervical squamous intraepithelial lesions: associations with lesion grade, state of the HPV16 E2 gene and clinical outcome

**DOI:** 10.1038/sj.bjc.6602237

**Published:** 2004-11-16

**Authors:** W Alazawi, M Pett, S Strauss, R Moseley, J Gray, M Stanley, N Coleman

**Affiliations:** 1 Medical Research Council Cancer Cell Unit, Hutchison/MRC Research Centre, Hills Road, Cambridge CB2 2XZ, UK; 2 Department of Pathology, University of Cambridge, Tennis Court Road, Cambridge CB2 1DQ, UK; 3 Health Protection Agency, Colindale Avenue, London NW9 5HT, UK; 4 Department of Histopathology, Addenbrooke's Hospital, Hills Road, Cambridge CB2 2QQ, UK

**Keywords:** CGH, papillomavirus, cervix, recurrence

## Abstract

Host genomic abnormalities may determine the natural history of cervical squamous intraepithelial lesions (SILs). We undertook comparative genomic hybridisation analysis of epithelium carefully microdissected from 70 cervical SILs, the largest series to date. In contrast to previous studies, we used frozen sections for optimal DNA quality and examined whether patterns of DNA copy number imbalance (CNI) are characteristic of SIL grade, human papillomavirus (HPV) status and postoperative recurrence. We identified more CNIs in cervical SIL than previously described, with more CNIs per case in high-grade squamous intraepithelial lesion (HG-SIL) than in low-grade squamous intraepithelial lesion (LG-SIL) (*P*=0.04). While some CNIs were seen at similar frequencies in HG-SIL and LG-SIL, others, including gain on 1q, 3q and 16q, were found frequently in HG-SIL but not in LG-SIL. There were significantly more CNIs per case in HG-SILs showing loss of the HPV16 E2 gene (a repressor of viral oncogene transcription) (*P*=0.026) and in HG-SILs that subsequently recurred (*P*=0.04). Our data are consistent with sequential acquisition of CNIs in cervical SIL progression. Higher frequency of CNI in association with E2 gene loss supports *in vitro* evidence that high-risk HPV integration is associated with genomic instability. Further investigation of the clinical value of specific host genomic abnormalities in cervical SIL is warranted.

Cervical squamous cell carcinoma (SCC) usually arises from a subset of high-grade squamous intraepithelial lesions (HG-SIL), which in turn are thought to arise from a subset of low-grade squamous intraepithelial lesions (LG-SIL) (Holowaty *et al*, 1999; Franco *et al*, 2001). Untreated SILs have the potential to regress, persist or progress, and up to 8.6% of SILs recur locally following complete excision (Mohamed-Noor *et al*, 1997; Hulman *et al*, 1998; Nagai *et al*, 2000; Chao *et al*, 2004). However, the molecular pathology of cervical SILs is poorly understood and it is currently not possible to predict the natural history of an individual lesion. Unnecessary follow-up of women with cervical SILs destined not to progress or recur imposes large burdens on colposcopy services worldwide and produces substantial adverse psychosocial consequences for individual patients. There is therefore a very important requirement for new rational and objective approaches to improving diagnosis and prediction of outcome in cervical SIL.

Infection with high-risk human papillomavirus (HR-HPV) is known to be an independent risk factor for progression of LG-SIL to HG-SIL and for the development of SCC (Remmink *et al*, 1995; Konno *et al*, 1998). However, HPV testing is inherently nonspecific at identifying patients destined to develop cervical SCC (Sasieni, 2000) or HG-SIL (Lorincz *et al*, 2002). Other biological factors of importance in the progression of SIL or recurrence after treatment include persistence of HR-HPV (Costa *et al*, 2003), integration of HR-HPV into host chromosomes and the acquisition of secondary host genomic abnormalities (Lazo, 1999). Integration frequently causes disruption of the HR-HPV E2 transcriptional repressor with consequent deregulation of HR-HPV oncogenes, events that we have recently demonstrated to be important in inducing high-level genomic instability in cervical keratinocytes *in vitro* (Pett *et al*, 2004).

Very little information is available as to whether particular host genomic abnormalities in cervical SIL may be characteristic of lesion grade, HPV status or clinical outcome. Allelic imbalances have been reported on numerous chromosome arms in cervical carcinomas and small numbers of SILs (Steenbergen *et al*, 1996; Chu *et al*, 1999; Lazo, 1999; Pulido *et al*, 2000), and there is some evidence of progressive accumulation of allelic imbalances with increasing grade of abnormality. (Larson *et al*, 1997; Chu *et al*, 1999; Luft *et al*, 1999; Chung *et al*, 2000; Lin *et al*, 2000; Chuaqui *et al*, 2001).

Likewise, data from comparative genomic hybridisation (CGH) have been reported for only relatively small numbers of cervical SILs (Heselmeyer *et al*, 1996; Kirchhoff *et al*, 1999, 2001; Umayahara *et al*, 2002). These studies have generally suggested that relatively few copy number imbalances (CNIs) exist in SIL. However, such data should be interpreted with caution, since all studies used formalin-fixed paraffin-embedded tissue, in which DNA degradation may cause difficulties with CGH, and cells microdissected under histological control do not appear to have been analysed in all studies (Kirchhoff *et al*, 1999, 2001). Moreover, one study combined cases equivalent to LG-SIL with cases equivalent to HG-SIL (Kirchhoff *et al*, 2001) and another examined SIL adjacent to SCC (Umayahara *et al*, 2002), which may not represent typical SIL. Most importantly, no study to date has correlated the number and type of CNIs in cervical SIL with key clinicopathological information such as HPV status and postsurgical outcome.

We have therefore performed CGH using metaphase chromosome targets to investigate the number and frequency of CNIs in a large series of 70 snap-frozen cervical SILs from 70 different patients, in which the abnormal epithelium was microdissected from serial frozen sections under histological control. None of the SILs was accompanied by SCC. We had three objectives when performing this work. Firstly, we aimed to characterise the number and locations of CNIs in cervical HG-SIL and LG-SIL. We analysed the stage of disease progression at which individual abnormalities occurred, in order to identify possible pathways of progression. We reasoned that any abnormalities present at similar frequencies in LG-SIL and HG-SIL might represent early events in the pathogenesis of SIL, while abnormalities present at a higher frequency in HG-SIL than in LG-SIL might reflect those involved in disease progression.

Secondly, we sought to identify any associations between CNI and the state of the HPV16 E2 gene in HPV16-positive HG-SILs. Initially, we performed HPV detection and typing on all cases using nested PCR and reverse line hybridisation. In HG-SILs containing HPV16, the HPV type most frequently associated with cervical SCC (Bosch *et al*, 1995), we examined associations between CNIs and the presence or absence of the HPV16 E2 and E7 genes, as detected by PCR. We aimed to identify cases in which HPV16 E2 was lost but HPV16 E7 was retained, as these cases would be expected to show derepression of HR-HPV oncogenes. Previous validation studies using Southern blotting had shown that such E2-negative/E7-positive cases contained HPV16 integrants in the absence of HPV16 episomes (Das *et al*, 1992). The E2/E7 PCR technique was employed, as the microdissected samples did not yield sufficient quantities of genomic DNA (gDNA) and RNA to enable us to use optimal techniques for assessment of HR-HPV integration, such as restriction site PCR (Thorland *et al*, 2000) or amplification of papillomavirus oncogene transcripts (Wentzensen *et al*, 2002). The third and final aim of our study was to assess the clinical value of selected host and viral parameters in predicting recurrence of HG-SIL after complete excision by large loop excision of the transformation zone (LLETZ).

## MATERIALS AND METHODS

### Tissue

**Figure 1:**
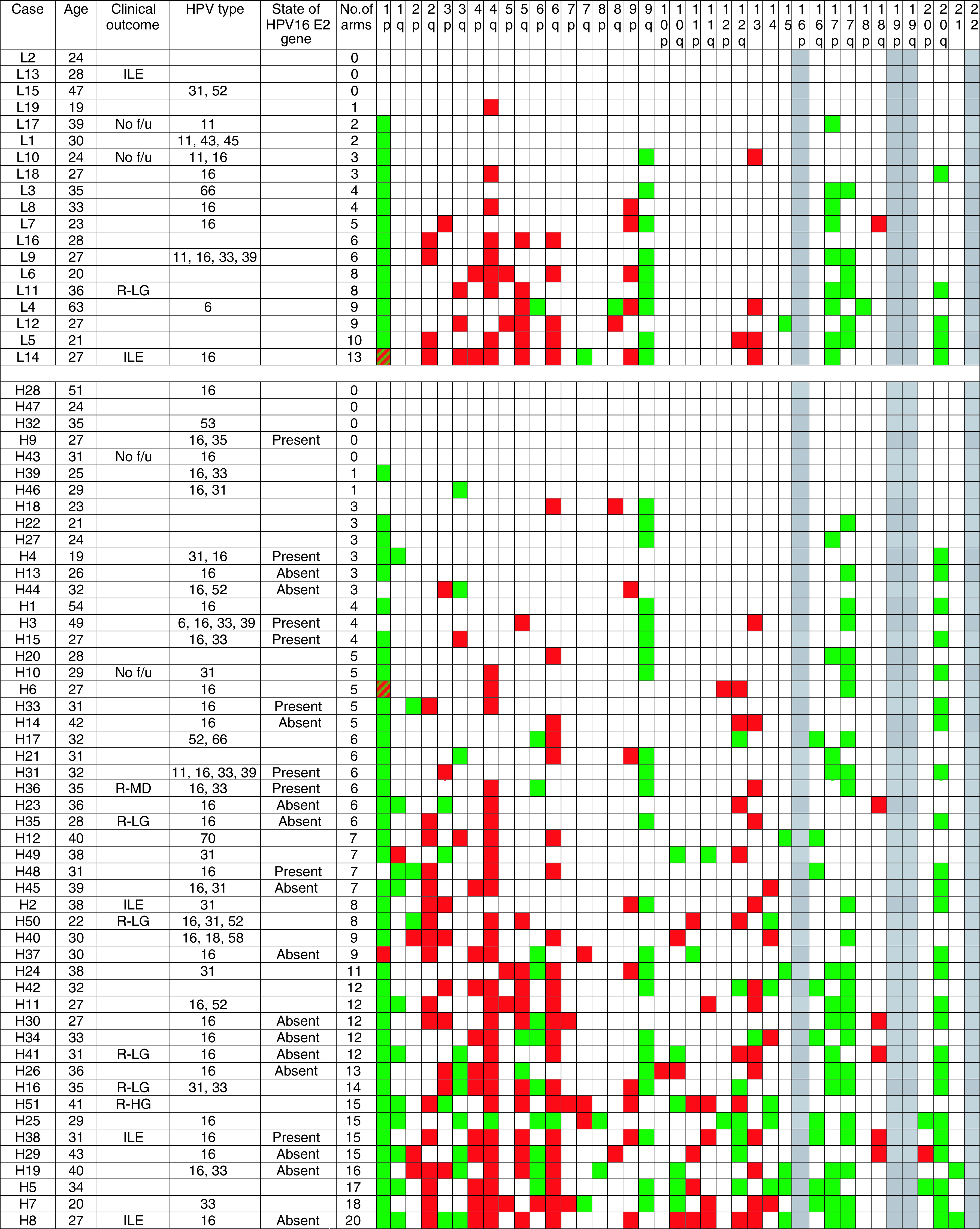
Clinicopathological data and frequency of CNIs in the 70 SILs studied. Prefix L denotes low-grade SIL and prefix H denotes high-grade SIL. Clinical outcome is shown if noteworthy. (ILE=inadequate local excision; No f/u=no follow-up data available; R-LG=recurred with a histological diagnosis of LG-SIL; R-HG=recurred with a histological diagnosis of HG-SIL; R-MiD=recurred with cytological mild dyskaryosis). Also listed are patient age; HPV types detected in the 53 cases analysed; the presence or absence of the HPV16 E2 gene in cases of HG-SIL that were HPV16 positive and tested (*n*=23); and the number of chromosome arms showing DNA CNI. Cases are sorted by a grade of SIL and then ranked by the number of arms showing CNI. For each arm, white boxes indicate no CNI, green boxes indicate gain, red boxes indicate loss and green/red striped boxes indicate gain and loss on the same arm. No data are shown for chromosomes 16p, 19 and 22, for which analysis by CGH is unreliable. In all 23 cases of HG-SIL tested for the state of the HPV16 E2 gene, the HPV16 E7 gene was detectable.

The experimental work was performed with the permission of the Cambridge Local Research Ethics Committee (Ref: 03/023). The study used gDNA extracted from SIL epithelium carefully microdissected from frozen sections of cervical tissue. The tissue was obtained from LLETZ samples removed by a consultant gynaecologist from 70 different patients undergoing treatment for cervical disease. None of the cases was associated with SCC. The LLETZ samples were placed on ice after removal and study tissue was removed and snap-frozen in liquid nitrogen within 30–60 min. Samples were numbered at the time of recruitment. The histopathological diagnosis in the frozen sections for microdissection was agreed by two consultant histopathologists. Of the 70 cases, 51 were HG-SIL (given the prefix H; Figure 1

) and 19 LG-SIL (given the prefix L). The median patient age was 30 years (range 19–63 years). Normal cervical epithelium and stroma were obtained from six hysterectomies performed for non-neoplastic disease unrelated to the cervix.

### Identification of disease recurrence

Cases of recurrence following complete local excision were identified from review of patient records by appropriate medical practitioners. The median follow-up period was 45 months (range 11–65 months), which is appropriate for assessment of local recurrence (Mohamed-Noor *et al*, 1997; Hulman *et al*, 1998; Nagai *et al*, 2000; Chao *et al*, 2004). Recurrence was defined as a diagnosis of SIL of either grade by histology or of dyskaryosis of any grade by cytology provided the original lesion was reported as completely excised and there had been at least one negative smear and/or biopsy between the original LLETZ and the recurrence. ‘Borderline’ cytological abnormalities were not regarded as recurrences. All samples were anonymised and researchers were blinded to the available clinical data until the CGH analysis was complete.

### Microdissection and DNA extraction

In all, 10 consecutive 10 *μ*m sections were cut from each frozen tissue block. The first and last were stained with haematoxylin and eosin (H&E) and used as guides for identifying SILs in the intervening unstained sections. The abnormal epithelium was microdissected and collected using sterile scalpels under stereoscopic visualisation in a microdissecting microscope at × 15–25 magnification. Some sections were stained by H&E following microdissection, in order to assess the accuracy of the procedure. In all cases, at least 80% of the microdissected tissue was composed of abnormal epithelium. The microdissected tissue was placed in 50 *μ*l of 10 mM Tris/1 mM EDTA (pH 8.0) buffer containing 0.4 *μ*g *μ*l^−1^ Proteinase K (Sigma, Poole UK) and incubated at 37°C overnight. Genomic DNA for HPV typing was extracted from lysates of microdissected cells using guanidinium isothiocyanate/silica as described (Boom *et al*, 1990).

### HPV typing

PCR for HPV detection was performed as described previously (Strauss *et al*, 1999) using the L1 consensus degenerate primers MY09 and MY11 for the initial amplification (expected product size 452 bp) followed by a second-round nested PCR using the GP5- and GP6-positive primers (expected product size 150 bp).

HPV typing was performed by reverse line hybridisation, as described elsewhere (Jordens *et al*, 2000), using probes complementary to sequences of the L1 region of HPV types 2, 6, 11, 16, 18, 31, 33, 35, 39, 41, 42, 43, 45, 50, 51, 52, 53, 54, 56, 58, 59, 61, 62, 66, 67, 70, 72, 81 and Han 831. HPV types 16, 18, 31, 33, 35, 39, 45, 51, 52, 56, 58, 59 and 66 were regarded as HR-HPV (Munoz *et al*, 2003), while the other types were regarded as low-risk HPV.

### State of HPV16 E2 gene

Lysates of microdissected cells from HPV16-positive cases (where available) were used to assess the presence or absence of the HPV16 E2 gene, with reference to the HPV16 E7 gene.

A measure of 5 *μ*l of lysate was PCR amplified using the AmpliTaq Gold kit (Applied Bioscience, Foster City, CA, USA): 25 mM MgCl_2_, 2 mM dNTPs, 5 U *μ*l^−1^ AmpliTaq Gold and 20 *μ*M primer pairs for either full-length HPV16 E2 (forward: 5′-TGCGATGGATCCATGGAGACTCTTTGCCAACG-3′; reverse: 5′-TGCGATGGATTCTCATATAGACATAAATCCAG-3′; expected product size 1139 bp) or full-length HPV16 E7 (forward 5′-ATGCATGGAGATACACCTAC-3′; reverse 5′-TGGTTTCTGAGAACAGATGGG-3′; expected product size 294 bp). HPV16 DNA in the pSPHPV-16 plasmid (Stanley *et al*, 1989) was used as a positive control.

Samples were considered positive for a particular reaction when a band of the appropriate size was clearly identified. Cases showing equivocal positivity were not encountered in this study. All amplifications were preformed in triplicate. On every occasion, the presence or absence of a PCR product band was consistent for all three replicates.

### Comparative genomic hybridisation

A measure of 5 *μ*l of lysate of microdissected cells was used in a primary degenerate oligonucleotide primed (DOP) PCR reaction as described previously (Roberts *et al*, 1999). Test probes were made by labelling 250 ng of primary DOP products in a secondary DOP reaction incorporating digoxigenin-11-dUTP (Boehringer Mannheim, Germany). Reference DNA was obtained from normal male peripheral blood lymphocytes and subjected to two rounds of DOP–PCR, using biotin-16-dUTP in the secondary labelling reaction (Boehringer Mannheim, Germany). The test and reference DNAs were sex mismatched in order to provide an internal control.

Probes were made by ethanol coprecipitating 500 ng of test and reference products together with 5 *μ*g of Cot-1 DNA (Roche Diagnostics, Lewes, UK). Hybridisation to normal male metaphase spreads (Vysis, Richmond, UK) was as described previously (Sanoudou *et al*, 2000; Roberts *et al*, 2001). The biotin and digoxigenin labels were detected using avidin-Cy3 (Amersham Pharmacia Biotech, Little Chalfont, UK) and antidigoxigenin fluorescein isothiocyanate (FITC)-conjugated Fab fragments (Roche Diagnostics, Lewes, UK), respectively (Roberts *et al*, 2001).

For each case, seven to 12 metaphases were captured on an Axioplan II epifluorescence microscope (Zeiss, Welwyn Garden City, UK) equipped with narrow bandpass filter blocks for DAPI, Cy3 and FITC, a Sensys charge-coupled device camera (Photometrics, Tucson, AZ, USA) and SmartCapture VP imaging software (Digital Scientific, Cambridge, UK). Images were assessed using Quips CGH Analysis and Interpretation software (Vysis, Richmond, UK). Nine normal–normal control hybridisations were performed using six test samples from microdissected normal ectocervical epithelium and three test samples from microdissected normal cervical stroma. Based on the results of these hybridisations, the green-to-red (test-to-reference) fluorescence intensity ratio thresholds were set to 0.85 for loss and 1.15 for gain.

Chromosomes 16p, 19 and 22 were excluded from the CGH analysis as they yield unreliable CGH data (Kallioniemi *et al*, 1994; Zitzelsberger *et al*, 1997). The telomere of chromosome 1p was not excluded, as the SIL samples showed DNA gain at this region rather than DNA loss (which can represent a spurious finding; Zitzelsberger *et al*, 1997), with no evidence of such gain in the normal–normal hybridisations.

### Statistical analysis

Differences in the number of CNIs per case between sample groups were compared using the Mann–Whitney *U*-test. Differences in the frequency of individual CNIs between sample groups were compared using the *χ*^2^ test.

## RESULTS

### Clinical follow-up data

Of the 70 SILs examined by CGH, five had been inadequately excised and follow-up data were not available for four (Figure 1). In total, 61 cases (46 HG-SIL; 15 LG-SIL) were therefore completely excised and accompanied by adequate follow-up data. The median follow-up time was 45 months (range 11–65 months). Seven cases recurred (11%), of which six were HG-SIL (H16, H35, H36, H41, H50 and H51) and one was LG-SIL (L11). Recurrence was diagnosed by histological examination in all of these cases, except H36 where it was diagnosed by cytology. The median time for recurrence of the HG-SILs was 15 months (range 11–22 months).

### HPV typing

Sufficient DNA was available for HPV testing following CGH in 53 of the 70 cases. HPV DNA was detected in all of these cases. In all, 15 different HPV types were detected and multiple infections were seen in 20 (38%) cases. HR-HPV types were detected in 39 of 42 (93%) of the testable HG-SILs and in eight of 11 (73%) of the testable LG-SILs (Figure 1). HPV16 was detected in 39 cases (33 HG-SIL, six LG-SIL), but only one case (an HG-SIL) was HPV18 positive.

### E2/E7 PCR

**Figure 2:**
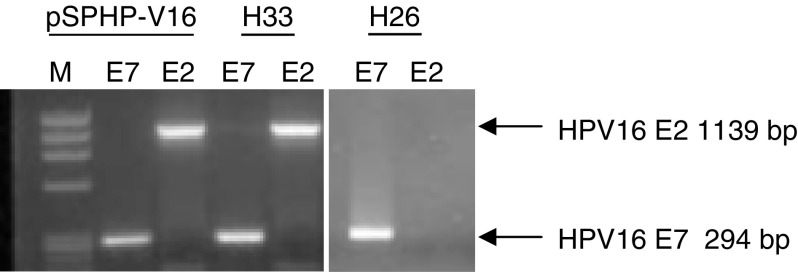
E2/E7 PCR in HPV16-positive HG-SILs. PCR for HPV16 E2 and HPV16 E7 genes in representative cases of HPV16-positive HG-SILs. M=ΦX DNA ladder (HT Biotechnologies Ltd, Cambridge, UK). The positive control pSPHPV-16 plasmid contains full-length HPV16. Whereas case H33 contains intact E7 and E2, H26 contains intact E7 but disrupted E2.

A total of 23 HPV16-positive HG-SILs had sufficient residual DNA to permit PCR assessment of the presence of the HPV16 E2 and E7 genes (Figure 2

). In total, 14 cases (61%) harboured HPV16 E7 but not HPV16 E2, consistent with integrated HPV16 in the absence of HPV16 episomes. Nine cases (39%) harboured intact HPV16 E2 and HPV16 E7 genes.

### Comparative genomic hybridisation

#### Associations with lesion grade

**Figure 3:**
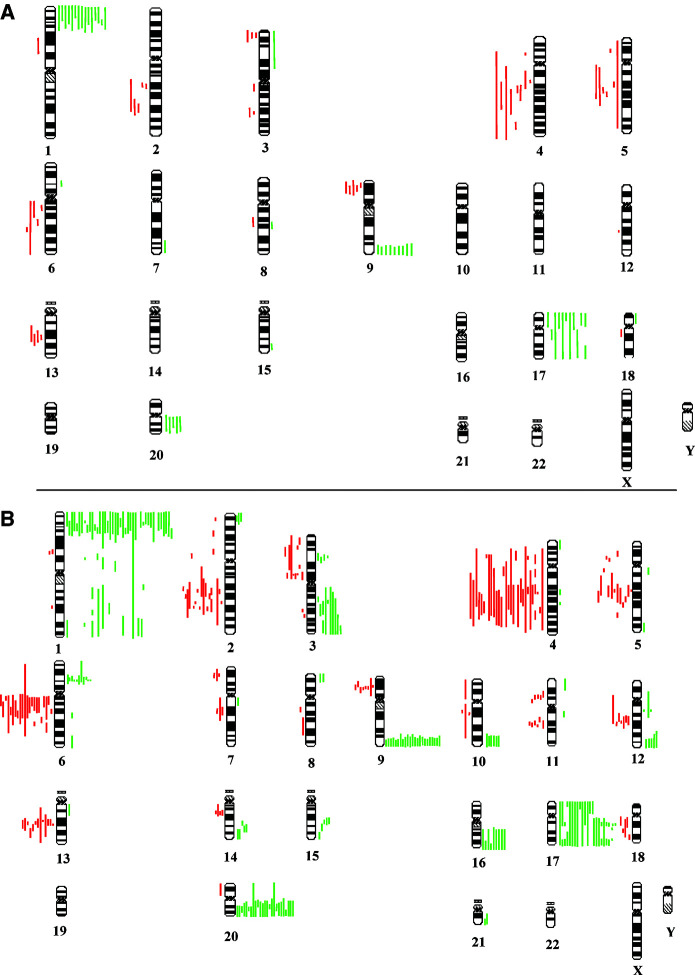
Summary copy number karyogram for 19 LG-SILs (**A**) and 51 HG-SILs (**B**). Each green bar to the right of a chromosome represents a region of DNA gain in a single case and each red bar to the left of a chromosome represents a region of DNA loss in a single case.

**Figure 4:**
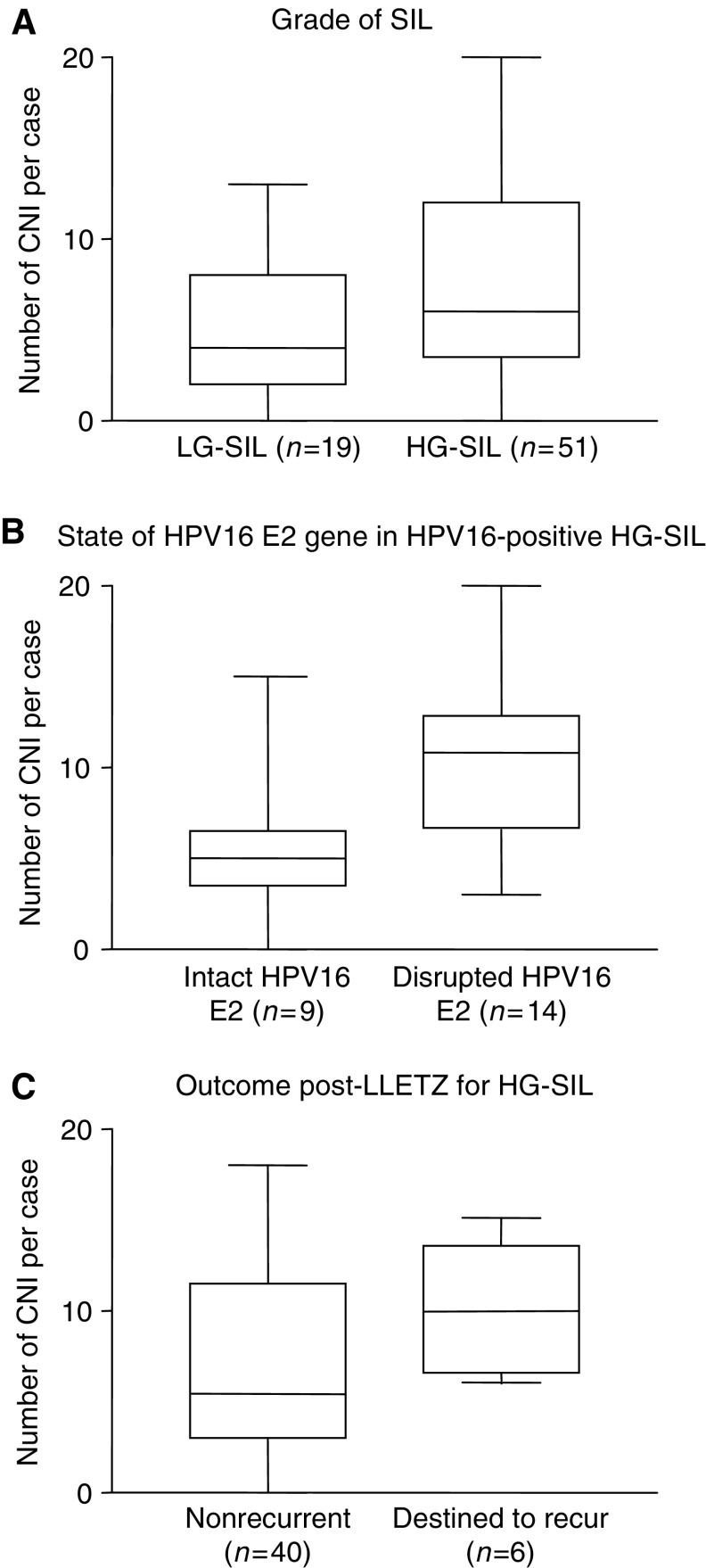
Number of CNIs per case according to selected clinicopathological features. Plots show the median (line), interquartile range (box) and full range (whiskers) of numbers of chromosome arms showing CNI per case in different groups of cases. There are significantly more CNIs per case in: (**A**) HG-SILs *vs* LG-SILs (*P*=0.04); (**B**) HPV16-positive HG-SIL with disrupted HPV16 E2 *vs* HPV16-positive HG-SIL with intact HPV16 E2 (*P*=0.026) and (**C**) HG-SIL destined to recur post-LLETZ *vs* HG-SIL that did not recur (*P*=0.04).

The summary of CGH copy number karyograms for all 70 cases examined are shown in Figure 3

and the number and locations of chromosome arms involved for each case is shown in Figure 1. There were more CNI per case in HG-SIL (median 6, range 0–20; *n*=51) than in LG-SIL (median 4, range 0–13; *n*=19) (*P*=0.04) (Figures 1 and 4a

).

The most frequently occurring abnormalities in the LG-SILs were gain on 1p (79%), 9q (47%), 17p (37%), 17q (32%) and 20q (32%) and loss on 4q (47%), 5q (32%), 2q (26%), 6q (26%), 9p (26%) and 13q (21%). The most frequently occurring abnormalities in the HG-SILs were gain on 1p (80%), 17q (47%), 20q (47%), 9q (45%) and 17p (27%) and loss on 4q (53%), 6q (43%), 2q (33%), 13q (25%) and 5q (24%).

Certain consistent regions of common gain and loss were identified, particularly gain at 1pter-1p32, 3q14–21, 6p21.3–21.2 and 9q34 and loss at 2q22–32, 4q22–28, 5q14–23, 6cen-q21, 9q21, 11q12–13, 11q14–21, 12q15–21 and 13q21–22. We also noted rarer gains at 3p21, 14q24 and 15q22 and rarer losses at 7p21, 7q21 and 14q12–13. No regions of amplification (test-to-reference fluorescence ratio >1.5) were seen in any case.

Figure 5

**Figure 5:**
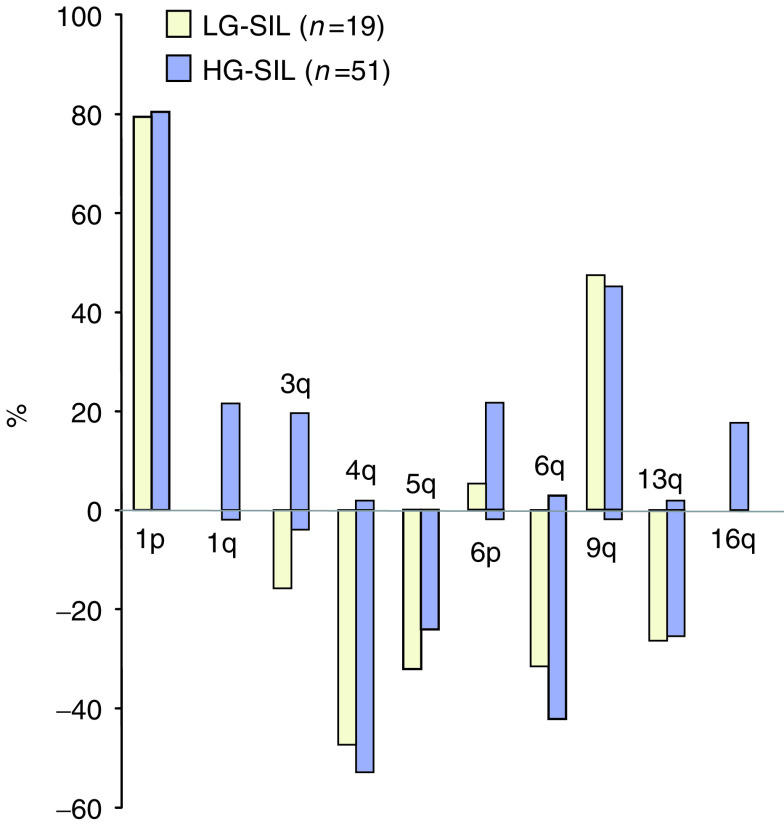
Frequency of selected CNIs in LG- and HG-SILs. Bars above the *x*-axis indicate the percentage frequency of gains on a chromosomal arm, and bars below the *x*-axis indicate the percentage frequency of losses on a chromosomal arm. Yellow bars=LG-SILs; blue bars=HG-SILs.

shows the frequency of gain and loss of selected chromosome arms in all cases examined. Some CNIs occurred at essentially similar frequencies in HG-SIL and LG-SIL, including gains on 1p and 9q and losses on 4q, 5q, 6q and 13q. In contrast, gains on 1q, 3q and 16q were found frequently in HG-SIL but not at all in LG-SIL (*P*<0.05 for each). Gain on 3q was seen in 10 cases of HG-SIL (20%) and was the only abnormality in one case. Gain of 6p was also seen frequently in HG-SIL (22%) but only in a single LG-SIL.

#### Associations with state of the HPV16 E2 gene in HPV16-positive HG-SIL

There were more CNIs per case in the 14 HPV16-positive HG-SIL cases with loss of the HPV16 E2 gene and retention of the HPV16 E7 gene (median 10.5, range 3–20), compared to the nine HPV16-positive HG-SIL cases with intact HPV16 E2 and HPV16 E7 (median 5, range 0–15) (*P*=0.026) (Figure 4b). The cases with loss of E2 also showed more frequent gain on 3q (*P*<0.05). On the other hand, we did observe high numbers of CNIs in some cases of HG-SIL in which the E2 gene was retained (e.g. case H38, which had 15 aberrant chromosome arms).

#### Associations with clinical outcome in HG-SIL

Of the 46 cases of completely excised HG-SIL with adequate follow-up data, there were more CNIs in the six cases that were destined to recur (median 10, range 6–15) than in the 40 that did not recur (median 5.5, range 0–18) (*P*=0.04) (Figure 4c). Loss of 4q (*P*=0.01) and loss of 5q (*P*<0.05) were more frequent in the HG-SILs destined to recur than in those that did not recur.

## DISCUSSION

We have identified more CNIs per case than previously reported in SIL. We observed a median of four CNIs per case in LG-SIL and six CNIs per case in HG-SIL, compared, for example, to a mean of 1.1 CNIs per case in lesions equivalent to LG-SIL and a mean of 4.1 CNIs per case in lesions equivalent to HG-SIL in a previous report (Umayahara *et al*, 2002). Our study has the advantages of using frozen sections of cervical SILs (unaccompanied by SCC) from which the lesional epithelium was microdissected, which may allow for greater sensitivity in detecting CNIs. On the other hand, it should be noted that the thresholds used for determination of copy number gain and loss have been greater in previous studies of cervical SIL (Heselmeyer *et al*, 1996; Kirchhoff *et al*, 1999; Umayahara *et al*, 2002).

Our data indicate that certain recurrent CNIs occur in both cervical LG-SIL and HG-SIL and suggest that there may also be sequential acquisition of imbalances during the progression from LG-SIL to HG-SIL. While the CNIs that occur at similar frequencies in LG-SIL and HG-SIL are consistent with early events, those that are significantly more frequent in HG-SIL may confer a selective advantage to LG-SIL cells and contribute to progression via clonal selection. Alternatively, the latter CNIs may represent an advantageous consequence of increased chromosomal instability in HG-SIL (Pett *et al*, 2004) and be of greater relevance in favouring subsequent progression to SCC.

CGH losses reported here are consistent with sites of allelic imbalance previously reported in cervical SIL and SCC (Lazo, 1999), including 3p12–3p14.1 (Larson *et al*, 1997; Chu *et al*, 1999; Chung *et al*, 2000), 4p16 (Larson *et al*, 1997), 4q21–q35 (Larson *et al*, 1997), 6p14–22 (Chatterjee *et al*, 2001; Steenbergen *et al*, 2001), 6q22–q27 (Chuaqui *et al*, 2001), 9p21 (Lin *et al*, 2000), 11p14–15 and 11q23 (Luft *et al*, 1999; Pulido *et al*, 2000). Further investigation of these sites of potential tumour suppressor genes is warranted.

We observed gain of 3q in 20% of 51 HG-SIL cases. Whereas this imbalance was initially described as defining the transition to SCC in the cervix (Heselmeyer *et al*, 1996), it was subsequently reported in eight of 37 (22%) paraffin-embedded ‘dysplastic’ lesions equivalent to HG-SIL (Kirchhoff *et al*, 1999; Kirchhoff *et al*, 2001), an observation that is supported by our data. Interestingly, we found that six of the 10 HG-SILs in our study that showed gain of 3q also showed loss on 3p or 13q, also supporting the previous suggestion of an association between these abnormalities (Kirchhoff *et al*, 1999). Our findings are consistent with the earlier identification of 3q25–q27 as the consensus region of gain.

Cases of HPV16-positive HG-SIL in which the E2 gene was disrupted showed significantly more CNIs and significantly more frequent gain on 3q than cases in which the E2 gene was intact. Data from the PCR approach that we used to detect E2 and E7 have previously been shown to correlate with the physical state of HPV in clinical specimens, as determined by Southern blotting (Das *et al*, 1992). Loss of E2 (with retention of E7) is consistent with the presence of integrated HPV16 in the absence of HPV16 episomes. The presence of the HPV16 E2 gene could either be due to episomal HPV16 or to integrated HPV16 in which the gene is retained, for example, following concatamerisation of the integrants. Our finding that the E2 gene was disrupted in 14 of 23 (61%) testable HG-SILs is consistent with previous PCR data (Tonon *et al*, 2001). Greater chromosomal instability following loss of the E2 gene is likely to be associated with derepression of the HPV16 oncogenes E7 and E6, which can cooperate in the induction of mitotic defects and genomic instability (Duensing *et al*, 2000; Plug-DeMaggio *et al*, 2004).

For some cases at least, loss of HR-HPV E2 may therefore be a significant intermediate step in cervical oncogenesis, occurring between infection with HR-HPV and the development of chromosomal instability. Indeed, these data support our recent *in vitro* observations that integration of HPV16 in cervical keratinocytes, with disruption of the E2 gene as is typically seen *in vivo*, is closely associated with the development of high-level genomic instability (Pett *et al*, 2004). On the other hand, our identification in the present study of cases of HG-SIL that retain the HPV16 E2 gene yet show numerous CNIs (e.g. case H38, with 15 aberrant arms) is consistent with the notion that loss of E2 function is not essential for chromosomal instability to develop in cervical keratinocytes. It should be noted, however, that the low cell yield provided by the microdissection approach that we chose to adopt did not enable us to assess levels of E2 gene expression in the samples that we examined. Moreover, because of clonal heterogeneity within SILs, cells containing the E2 gene may have been different to those showing numerous CNIs.

Of the 46 cases of HG-SIL in our study with histologically clear excision margins and adequate follow-up data, six (13%) recurred. This rate of local recurrence is broadly similar to that described by other authors (Hulman *et al*, 1998; Nagai *et al*, 2000). HG-SILs destined to recur showed significantly more CNIs and significantly more frequent loss of 4q and 5q than HG-SILs that did not recur. Our observations were made on relatively small numbers of cases, using a whole genome approach that would not be appropriate for routine clinical use. However, our data support the notion that detection of one or more specific host cell abnormalities (either alone or in combination with tests of HPV type, physical state, etc.) may ultimately prove to be of value in risk stratification and clinical management of cervical SILs.

It is likely that shared risk factors underlie the association that we have observed between the number of CNIs in HG-SIL and lesional recurrence despite complete local excision. Most important may be viral factors, such as persistence and integration of HR-HPV (Cuzick, 1997; Cruickshank *et al*, 2002). However, there may also be a role for host genetic polymorphisms, such as those affecting *TP53* (Klug *et al*, 2001) or local host immunity (Cuzick *et al*, 2000; Maciag *et al*, 2000), and/or environmental factors such as smoking, local hormonal effects and other sexually transmitted diseases (Kjellberg *et al*, 2000).

In summary, we have obtained molecular cytogenetic data that are consistent with a model of sequential acquisition of genomic CNIs in the development and progression of cervical SIL. We show that a high number of CNIs, as well as particular sites of imbalance, are associated with loss of the HPV16 E2 gene in HPV16-positive HG-SIL and with recurrence of HG-SIL after adequate local excision. It may ultimately be possible to improve the prediction of postsurgical outcome in SIL by assessing specific genomic abnormalities in the excised lesion. Our findings now require validation in a different, larger group of cases, using higher resolution approaches.
